# Impact Compression Test and Numerical Simulation Analysis of Concrete after Thermal Treatment in Complex Stress State

**DOI:** 10.3390/ma12121938

**Published:** 2019-06-16

**Authors:** Yue Zhai, Yubai Li, Yan Li, Yunsheng Zhang, Fandong Meng, Ming Lu

**Affiliations:** 1 School of Geology Engineering and Geomatics, Chang’an University, Xi’an 710054, China; zy@chd.edu.cn (Y.Z.); liyanlwbdlp@chd.edu.cn (Y.L.); zhangyunsheng@chd.edu.cn (Y.Z.); mengfandong@chd.edu.cn (F.M.); ming.lu@sintef.no (M.L.); 2 School of Earth Sciences and Resources, China University of Geosciences (Beijing), Beijing 100083, China

**Keywords:** concrete, thermal treatment, impact compression, passive confining pressure, fracture characteristics

## Abstract

To study the dynamic mechanical properties and fracture law of concrete after thermal treatment and reveal its mechanism, the impact compression test was carried out on different thermal-treated (400–800 °C) concrete specimens using a split Hopkinson pressure bas (SHPB) system. By using ANSYS/LS-DYNA, the finite element numerical simulation of the test process was illustrated. The research showed that under passive confining pressure, the more the loading rate is increased, the more obvious the effect of the passive confining pressure on the concrete specimen, as well as the more significant the improvement of the peak stress compared with the uniaxial test. On the other hand, as the temperature damage effect is enhanced, the increase in the material strength at different loading rates is reduced. Numerical simulations showed that in a uniaxial test, as the impact rate increases, the crack initiation time advances, and the degree of fracture increases at the same rate as that of the loading time. In the case of confining pressure, the stress gradually decreases to the edge from the center, and has a significant circumferential diffusion characteristic. The circumferential restraint of the passive confining pressure limits the radial deformation ability of the material to a certain extent, thereby increasing the axial compressive strength. In the analysis of the crushing process of concrete specimens, it was found that the fracture form showed a strong rate dependence. When the loading rate is low, the fracture form is a cleavage-like failure. As the loading rate increases, the fracture form changes to crush failure. The research results provide the necessary theoretical basis for the safety assessment, reinforcement, and maintenance of concrete structures after fire.

## 1. Introduction

As a widely used economical and practical building material, concrete plays an important role in civil construction, transportation and water conservancy construction, and underground engineering construction. Whether for civil engineering or national defense engineering, the concrete structure will be subjected to a quasi-static load, that is, a normal design load. Moreover, concrete structures are often subjected to violent dynamic loads such as impact loads or blast loads. In the safety assessment, reinforcement, and maintenance of concrete structures after fire or explosion, it is necessary to master the dynamic mechanical response law and fracture law of concrete after high temperature treatment in a complex stress state. Therefore, it is particularly important to carry out in-depth research on this. However, concrete materials are typical heterogeneous brittle materials, including cement colloids, coarse and fine aggregates, and other microcracks, holes, weak media, etc., and materials are often affected by many external factors, such as temperature, moisture content, confining pressure, loading rate, etc. In addition, the impact loading test and analysis methods considering the confining pressure are limited. Therefore, there are still many problems to be solved in the study of the influence of multiple factors on the performance of concrete materials.

At present, the test system used to study the dynamic mechanical properties of materials mainly includes split Hopkinson pressure bar (SHPB), the drop-weight test, and other test systems. Among them, the SHPB test is the most widely used [1]. Gong [2,3] first used SHPB technology to study the dynamic strength of concrete materials. Brara [4] proposed the corresponding failure criteria. Numerous researchers have also conducted a large number of studies on the effectiveness of the SHPB test, such as the dispersion effect between the elastic pressure bar and the specimen and the inertial effect of the specimen during the test [5,6], the friction effect between the pressure bar and the specimen and the matching degree of the cross-section end face [7], as well as the ultra-high strain rate test technique [8].

The conventional SHPB test device needs to be modified for use in triaxial impact compression tests. Christensen et al. [9] improved the SHPB test device and designed a triaxial SHPB test device for rock materials, which realized the dynamic loading of rock materials under confining pressure. Gong et al. [10] studied the effects of high strain rates and low confining pressures on the dynamic mechanical properties of sandstone, and obtained the results that under approximately the same strain rate, the dynamic triaxial compressive strength linearly increases with the confining pressure. Yeakley et al. [11] studied the dynamic mechanical properties of basalt at a strain rate of 103 s^−1^ and a confining pressure of 69 MPa. The experimental results showed that the dynamic strength of basalt increases by 30% compared with quasi-static strength under confining pressure. Rome and Nemat-Nassei [12] improved and completed a set of triaxial SHPB test devices. Through the triaxial impact compression loading test of concrete materials, it was verified that the test device can be applied to concrete materials. Gerstle [13] used the test method to study the strength and deformation of concrete under multiaxial stress conditions, and summarized the concrete test results obtained by many testers under complex stress conditions, which is of great significance. Mier [14] studied the response of concrete under a triaxial stress state through experimental methods, and found by crack detection technology that the anisotropic stress–strain behavior was caused by the development of weak regions in larger particle aggregation regions during concrete hardening. The difference observed in macroscopic stress–strain behavior was the result of different crack distribution directions. Salami [15] carried out a relevant experimental analysis on the deformation characteristics of concrete specimens with multiaxial stress states under different loading rates, and further expanded the field of concrete materials in the experimental research direction. With the continuous development of test methods and techniques, the research on the dynamic mechanical properties of brittle materials such as concrete under complex stress conditions is also deepening.

However, the SHPB experiment can only study the macroscopic mechanical properties of the material. The microscopic laws of the stress, strain distribution, and crack propagation of the specimen need to be studied by numerical simulation. Bertholf and Karnes [16] used two-dimensional numerical analysis to describe the effects of friction, geometry, and strain rate on the test. Zhu [17] analyzed the failure process of dynamically loaded rock media by simulating the basic principle of the failure process of rock under static and dynamic loads. Besides, the effects of axial static stress and dynamic stress on rock damage and failure process are also studied. Guo [18] analyzed the temperature-dependent changes of peak stress, peak strain, and elastic modulus of materials under passive confining pressure by numerical simulation. Park [19] used the finite element method to analyze the dynamic response of concrete and mortar under high strain rates, and discussed the influence of aggregate volume fractions on the dynamic bearing capacity of concrete. The numerical simulation also showed that as the aggregate integral number increased, the energy absorption increases, and energy dissipation occurs with deformation. Zhang [20] used the HJC (Holmquist-Johnson-Cook) model to simulate the SHPB impact test with confining pressure for concrete, and it indicated that confining pressure increases with the growth of strength, ductility, and toughness.

The current research in this area is mainly for the uniaxial test and numerical analysis of the room temperature specimens. The comparative study on the passive confining pressure test and numerical simulation of the concrete after thermal treatment is relatively rare, and at the same time, there have been relatively few studies on the specimen crushing process and crushing law. Therefore, this study uses the combination of experiment and numerical simulation to analyze the impact mechanical properties and dynamic failure of different loading rates under the passive confining pressure of concrete after thermal treatment. 

## 2. Materials and Methods

### 2.1. Thermal Treatment Concrete Specimen Preparation

Concrete specimens with a uniaxial compressive strength of 35 MPa are most commonly used as test materials. Its main ingredients are common Portland cement, pebbles with diameters of 5 to 20 mm, medium sand (containing about 10% of the mud), fly ash, mineral powder (steel slag powder), and other additives (water reducing admixture). The specific mixing ratios and gradation ranges are shown in Table 1. The concrete material was cast into a cylinder 200 mm in height and 98 mm in diameter, and cured in a standard curing room for 28 days. The test specimens were processed into a 50 mm (height) × 98 mm (diameter) cylinder with parallel and smooth ends. 

The temperature-controlled heating device used in the experiment is an artificial intelligence temperature measuring and controlling device of the AI-518 model (Sigma Furnace Industry Co., Ltd., Luoyang, China). The effective size of the furnace was 400 × 200 × 160 mm^3^, and the maximum heating temperature was 1300 °C. Since the concrete strength does not change significantly within 400 °C [22], the target temperatures for this research were 400 °C, 600 °C, and 800 °C. In order to ensure that the specimen was heated evenly during the heating process, a slow temperature rise (4 °C/min) was adopted, and the specimen was stored at the target temperature for a while. The voltage during the preheating period was set to 75 V and the preheating time was 10 min; then, the voltage was raised to 100 V and heated to the target temperature. In order to ensure the consistency of the temperature inside and outside the specimen, the samples after heating at a constant temperature for two hours were taken out and placed at room temperature under natural ventilation for cooling.

### 2.2. SHPB Equipment 

**Figure 1:**
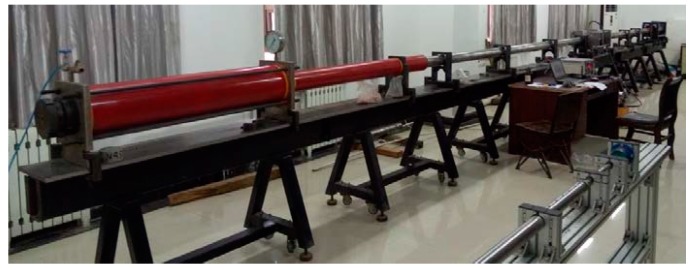
Split Hopkinson pressure bas (SHPB) equipment.

The compression bar of this experiment apparatus is shown in Figure 1.

In order to reduce the influence of the stress wave on the steep slope and the high-frequency oscillation at the peak, after several tests, a copper disc with a thickness of 1.5 mm and a diameter of 40 mm was determined as a waveform shaper [23]. 

**Figure 2:**
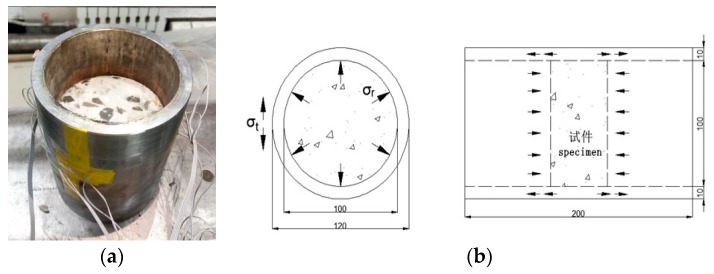
Passive confining pressure test device. (**a**) In-kind shooting; (**b**) Schematic diagram.

The passive confining device was made of No. 45 steel and was made into a sleeve with an inner diameter of 100 mm, an outer diameter of 120 mm, and a height of 200 mm (see Figure 2). The modulus of elasticity of the steel E was 210 GPa, the tensile strength $\sigma_{b}$ was 600 MPa, and the yield strength was $\sigma_{s}$ 335 MPa. The concrete specimen was radially expanded and deformed during axial impact compression, but this deformation was constrained by the presence of the steel sleeve, thereby generating a passive confining action. The length of the sleeve in the test was longer than the concrete specimen mainly because of two aspects: one was for the convenience of the test operation; the other was to prevent the incident bar from colliding with the sleeve during the impact compression process.

Two channels for collecting pulse signals are added to the sidewall of the passive confining sleeve, which is for measuring the hoop strain on the outside of the sleeve. According to the solution method of elastic mechanics, the friction between the sleeve and specimen can be neglected. The confining pressure value can be calculated by the measured value of the strain gauge on the sleeve outer wall. The calculation equation is as follows [24]:

(1) $$\sigma =\frac{\left(b^{2}-a^{2}\right)}{2a^{2}}\times E\times \varepsilon$$

where a is the inner diameter of the sleeve; b is the outer diameter of the sleeve; E is the elastic modulus of the sleeve; and ε is circumferential strain of the outer surface of the sleeve.

## 3. SHPB Test Results and Analysis 

### 3.1. Impact Compression Test

The control air pressures in the uniaxial and passive confining tests were 0.35 MPa and 0.5 MPa, resulting in initial impact loading rates of 14.23 m/s and 18.36 m/s, respectively. In the impact compression test with confining pressure, the confining pressure at the loading rate of 18.36 m/s was much larger than the confining pressure at the loading rate of 14.23 m/s. This phenomenon is particularly evident in the specimens after thermal treatment at 600 °C and 800 °C, and the maximum increase in confining pressure was 4.3 MPa. This is because as the loading rate increases, the axial compression of the specimen increases, resulting in an increase of the amount of radial deformation; therefore, it enhanced the circumferential restraint effect as well as significantly increased the passive confining pressure value. When the loading rate was the same, the confining pressure value did not increase or decrease with the change of heating temperature, indicating that the heating temperature has no obvious influence on the passive confining pressure value. See Table 2 for details.

**Figure 3:**
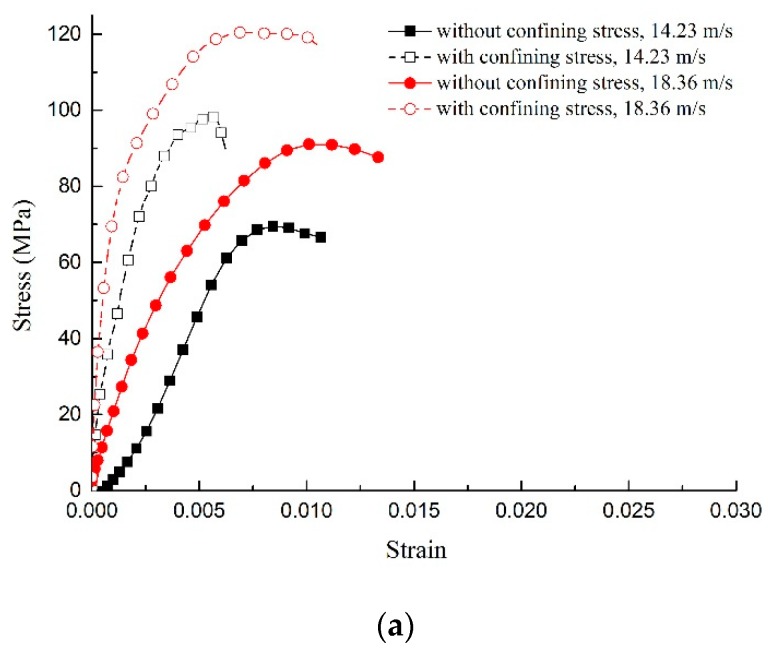

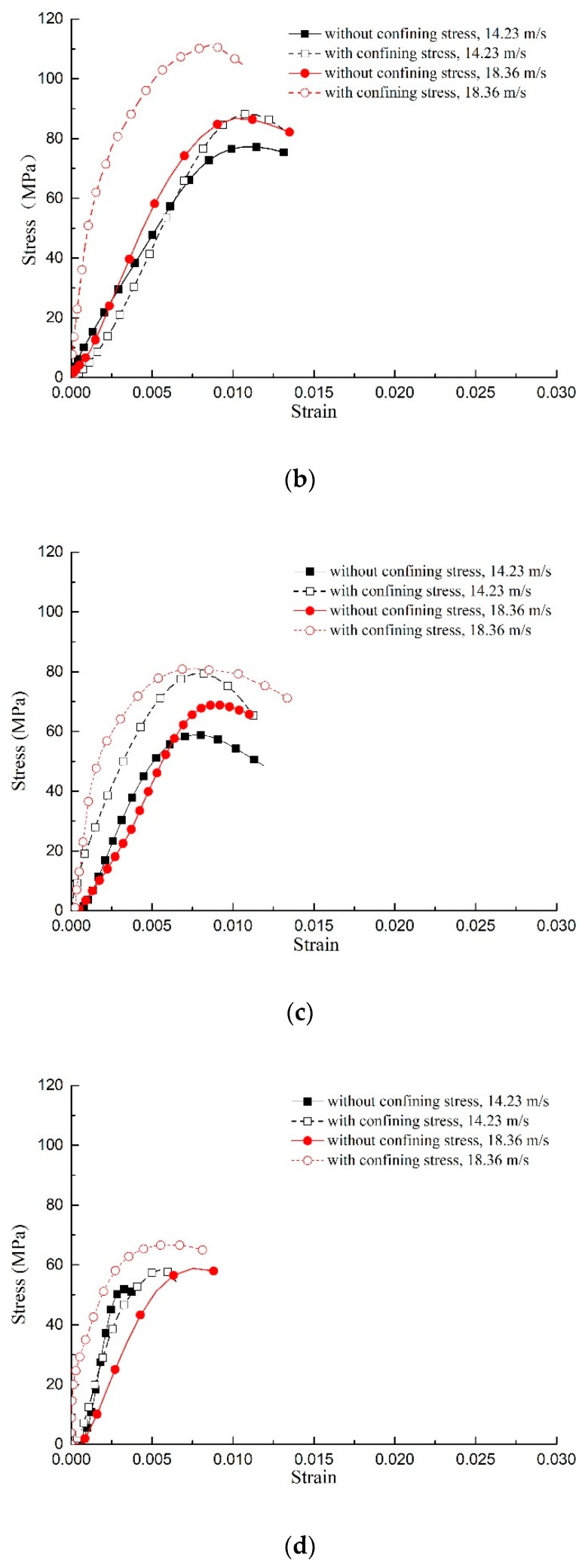
Stress–strain curves under different test conditions: (**a**) 20 °C; (**b**) 400 °C; (**c**) 600 °C; and (**d**) 800 °C.

The stress–strain curve of concrete material drawn by the uniaxial impact compression test and confining impact compression test data is shown in Figure 3. Compared with the uniaxial impact compression test, the peak stress under passive confining pressure was significantly enhanced. When the impact loading rate was 14.23 m/s, the peak stress under different temperatures was increased 12% to 41%, and when the impact loading rate was 18.36 m/s, the peak stress under different temperatures was increased 13% to 32%. It can be seen that the sleeve effect of the passive confining pressure constrained the radial deformation of specimen, thereby improving the brittleness characteristics, so the confining pressure significantly improved the impact resistance and ductility of the concrete. On the other hand, as the heating temperature increased and the damage effect was enhanced, the reinforcing effect of the confining pressure was weakened, so that the increase in the strength of the material was reduced.

### 3.2. Analysis of Crushing Concrete Specimen 

**Figure 4:**
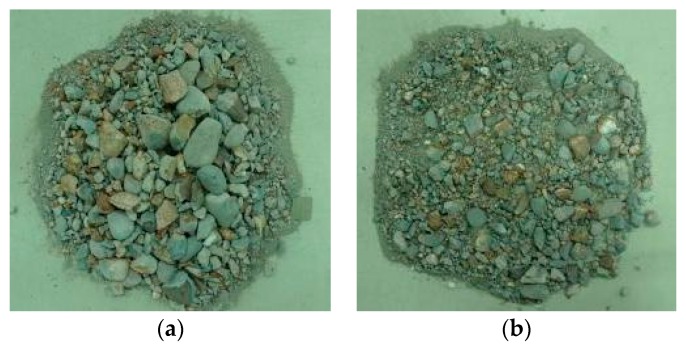
Concrete material fragments at different loading rates. (**a**) 14.23 m/s; (**b**) 18.36 m/s.

The uniaxial impact compression test shows that as the loading rate increased, the diameter of the fragments became smaller and smaller, and more dust was generated; especially when the temperature reached 600 °C and 800 °C, the compression damage of the specimen was very significant. Take a specimen heated at 600 °C as an example, as shown in Figure 4.

**Figure 5:**
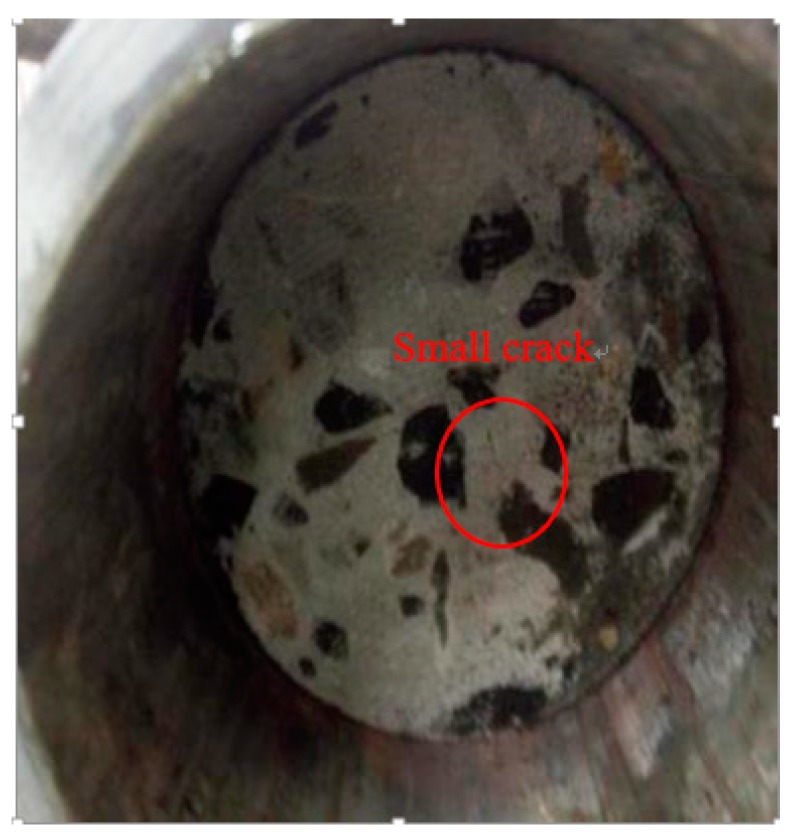
Picture of concrete specimen under impact compression with passive confining pressure (18.36 m/s, 600 °C).

The passive confining test shows that under the single test, the normal temperature specimens and specimens heated at 400 °C were intact; no obvious cracks appeared on the surface, and there was no obvious damage at the contact part between the steel sleeve and the concrete specimen. Concrete specimens treated at 600 °C and 800 °C are relatively complete. Small cracks appear on the surface of the specimen, but this was not obvious, as shown in Figure 5.

## 4. Numerical Simulation and Analysis of Concrete Fracture Mode

In order to better analyze the fracture characteristics and mechanism of concrete specimens, the above SHPB test was numerically simulated by ANSYS/LS-DYNA finite element software (ANSYS 19.0, ANSYS Inc., Canonsburg, PA, USA). Through the comparative analysis of the stress–strain curves obtained by the test and numerical simulation, the parameters of the HJC model used were calibrated to prove the feasibility of the establishment of the numerical model and the selection of material parameters. Based on this, the crack initiation time, crack development process, stress–strain redistribution, and fracture characteristics of concrete specimens under different temperatures and loading rates were analyzed.

### 4.1. Model Calibration

#### 4.1.1. HJC Model 

The HJC model is used in ANSYS/LS-DYNA finite element software. The model considers the damage of the material, the strain rate effect, and the effect of hydrostatic pressure on the yield stress. The HJC model includes three aspects: a strength model, a damage model, and an equation of state [25].

**Figure 6:**
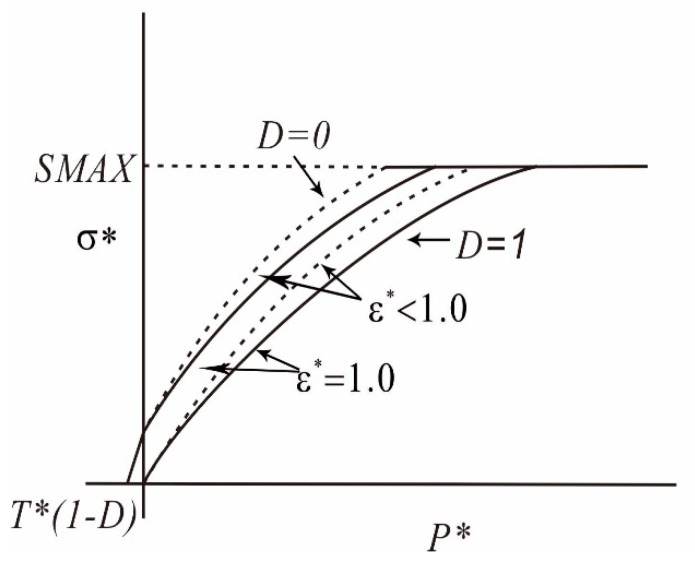
Strength model [26].

(1) The expression of the yield surface equation of the HJC model (Figure 6) is:

(2) $$\sigma^{*}=[A(1-D)+BP^{*N}](1+Cln{\dot{\varepsilon }}^{*})$$

where $\sigma^{*}=\sigma /f_{c}$ is the normalized equivalent stress, $\sigma^{*}\leq \mathrm{SMAX}$ σ is the actual equivalent stress, and $f_{c}$ is the quasi-static uniaxial compressive strength. *A* is the normalized cohesive strength, *B* is the normalized pressure hardening coefficient, *N* is the pressure hardening exponent, and *SMAX* is the normalized maximum strength. *C* is the strain rate coefficient, and *D* is the damage variable (0≤D≤1). $P^{*}=P/f_{c}$ is the normalized pressure (where *P* is the actual pressure), and ${\dot{\varepsilon }}^{*}=\dot{\varepsilon }/{\dot{\varepsilon }}_{0}$ is the dimensionless strain rate (where $\dot{\varepsilon }$ is the actual strain rate and ${\dot{\varepsilon }}_{0}=1.0\;s^{-1}$ is the reference strain rate). 

**Figure 7:**
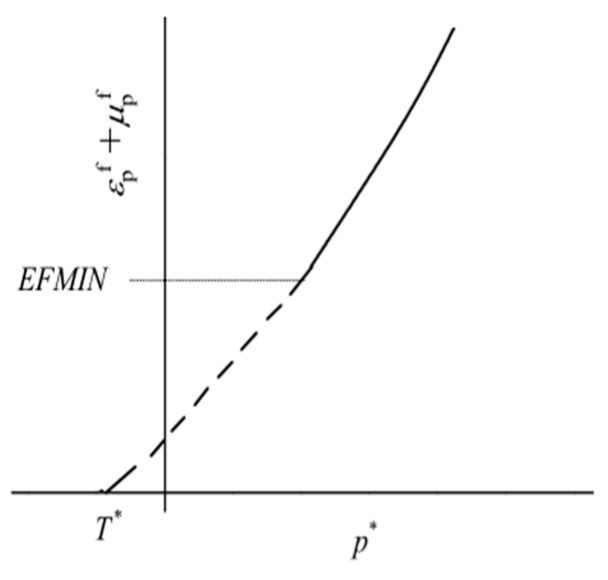
Damage model. [26].

(2) The HJC damage model is shown in Figure 7. The model accumulates damage from both equivalent plastic strain and plastic volumetric strain, and is expressed as:

(3) $$D=\sum \frac{\Delta \varepsilon_{P}+\Delta \mu_{P}}{\varepsilon_{p}^{f}+\mu_{p}^{f}}=\sum \frac{\Delta \varepsilon_{P}+\Delta \mu_{P}}{D_{1}{\left(P^{*}+T^{*}\right)}^{D_{2}}}$$

where $\Delta \varepsilon_{p}$ and $\Delta \mu_{p}$ are the equivalent plastic strain and plastic volumetric strain, respectively, during a cycle of integration. $D_{1}$ and $D_{2}$ are constants. $\varepsilon_{p}^{f}+\mu_{p}^{f}$ is the plastic strain to fracture under a constant pressure, P. $T^{*}=T/f_{c}$ is the normalized maximum tensile hydrostatic pressure (where T is the maximum tensile hydrostatic pressure that the material can withstand). *EFMIN* is the finite amount of plastic strain to fracture the material, and:

(4) $$D_{1}{\left(P^{*}+T^{*}\right)}^{D_{2}}\geq \mathrm{EFMIN}$$

**Figure 8:**
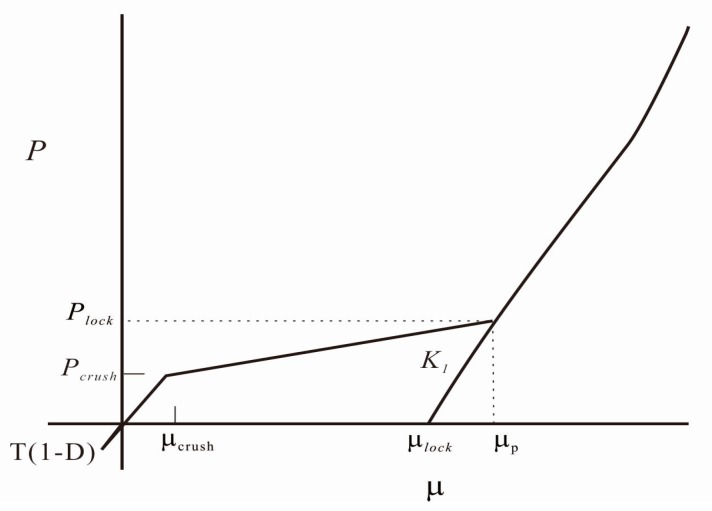
Equation of state [26].

(3) The HJC model equation of state is shown in Figure 8. The equation is divided into three phases: a linear elastic phase, a plastic phase, and a compaction phase.

The first region is linear elastic (0 ≤ *μ* ≤ $\mu_{crush}$):

(5) P=Kμ

where *P* is the hydrostatic pressure, $K=P_{crush}/\mu_{crush}$ is the elastic bulk modulus (where $P_{crush}$ and $\mu_{crush}$ are the pressure and volumetric strain that occur in a uniaxial stress compression test), and μ is the standard volumetric strain. 

The second region is defined as transition ($\mu_{crush}$ ≤ *μ* ≤ $\mu_{lock}$); the pores in the concrete material are compressed and plastically deformed:

(6) $$P=P_{crush}+K_{lock}\left(\mu -\mu_{crush}\right)\;(\mathrm{loading})$$

where $K_{lock}=\left(P_{lock}-P_{crush}\right)/\left(\mu_{lock}-\mu_{crush}\right)$, $P_{crush}$, and $\mu_{crush}$ are the pressure and volumetric strain that occur in a uniaxial stress compression test.

(7) $$P=P_{crush}+K_{lock}\left(\mu_{0}-\mu_{crush}\right)+\left[\left(1-F\right)K+FK_{lock}\right]\left(\mu -\mu_{0}\right)\;(\mathrm{unloading})$$

where $F=\left(\mu_{0}-\mu_{crush}\right)/\left(\mu_{lock}-\mu_{crush}\right)$, and $\mu_{0}$ is the volumetric strain before unloading. 

The third region is defined as compaction ($\mu \geq \mu_{lock}$). The internal pores of the material are completely dense. At this stage, the material is completely broken. Usually, the loading and unloading equations are expressed as:

(8) $$P=K_{1}\bar{\mu }+K_{2}{\bar{\mu }}^{2}+K_{3}{\bar{\mu }}^{3}\;(\mathrm{loading})$$

(9) $$P=K_{1}\bar{\mu }\;(\mathrm{unloading})$$

where $\bar{\mu }=\left(\mu -\mu_{lock}\right)/\left(1+\mu_{lock}\right)$ is the modified volumetric strain, and $K_{1}$, $K_{2}$, and $K_{3}$ are constants. In order to prevent the softening effect that the material may cause just after entering the compaction zone of the third stage, it is introduced as $\bar{\mu }$.

#### 4.1.2. Parameter Selection

As can be seen from the previous section, the HJC model contains 21 parameters, including the type of failure. In numerical simulation, the model parameters will directly affect the accuracy of the results.

##### Determination of $\rho_{0}$, $f_{c}$, T, and G

$\rho_{0}$ (concrete density) and $f_{c}$ can be measured by experiment. *T* is calculated according to the relationship proposed by:

(10) $$T=0.62{\left(f_{c}\right)}^{1/2}$$

The expression of *G* (shear modulus) is:

(11) G=E/2(1+v)

where *E* is the elastic modulus and v is the Poisson’s ratio, both of which are obtained by static test in the laboratory. The parameter values after different high-temperature treatment are shown in Table 3:

##### Determination of $P_{crush}$, $P_{lock}$, $\mu_{crush}$, $\mu_{lock}$, $K_{1}$, $K_{2}$, and $K_{3}$

$P_{crush}$ can be obtained by Equation (12):

(12) $$P_{crush}=f_{c}/3$$

$\mu_{crush}$ can be obtained by Equation (13):

(13) $$\mu_{crush}=P_{crush}/K$$

where K is the bulk modulus of concrete.

$\mu_{lock}$ can be obtained by Equation (14):

(14) $$\mu_{lock}=\frac{\rho_{grain}}{\rho_{0}}-1$$

where ρ is the current density and $\rho_{0}$ is the initial density. 

The value of $P_{lock}$, and $K_{1}$, $K_{2}$, and $K_{3}$ refer to Ref. 26. The parameter values after different high-temperature treatment are shown in Table 4:

##### Determination of C, SMAX, EFMIN, $D_{1}$, and $D_{2}$

The value of *C*, *SMAX*, *EFMIN*, and $D_{2}$ can be obtained from Ref. 25. The value of $D_{1}$ is calculated by:

(15) $$D_{1}=0.01/\left(\frac{1}{6}+T^{*}\right)$$

The parameter values after different high-temperature treatment are shown in Table 5:

##### Dtermination of *A*, *B*, and *N*

*A* is a scale factor of the damage term. The value of *A* controls the proportion of the damage term in the yield surface equation, and its value has a large influence on the peak stress of the stress–strain curve. The value of *B* affects the proportion of the hydrostatic pressure term in the yield surface equation. Its value has little effect on the overall shape of the stress–strain curve, but it has a certain influence on the peak stress. The value of *N* will change the shape of the yielding section of the curve. Therefore, the above three parameters will be inverted according to the stress–strain curve (Figure 3) obtained from the dynamic compression test, so as to obtain the simulated parameter values under different working conditions.

#### 4.1.3. Numerical Simulation of Uniaxial Impact Compression

Table 6 gives the error range of the peak stress and strain obtained by the numerical simulation and the experiments under different loading rates. The peak stress error is between −1.0% and 11.4%, and the peak strain error is between −4.0% and 12.5%. It can be seen from the calculation results that the numerical simulation results are in good agreement with the experimental results. This conclusion is consistent with Ref. [27].

#### 4.1.4. Numerical Simulation of Passive Confining Pressure Impact Compression

**Figure 9:**
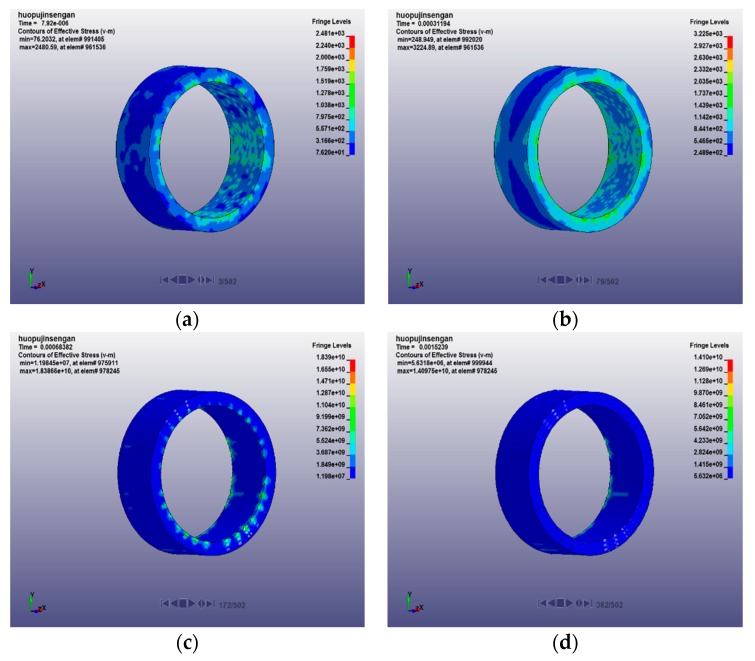
Propagation process of stress in the sleeve under passive confining pressure: (**a**) t = 7.92 μs; (**b**) t = 311.94 μs; (**c**) t = 683.82 μs; and (**d**) t = 1523.90 μs.

The axial propagation of stress in the sleeve under passive confining pressure is shown in Figure 9. It can be seen from the stress cloud diagram that the radial force of the specimen begins to gradually act on the sleeve at the initial stage of loading, but at this time, the sleeve is less affected by the concrete specimen. With the passage of time, the propagation of stress along the direction of the casing wall presents a more uniform annular distribution, the hoop restraint effect of the sleeve on the specimen becomes more and more obvious, and the edge stress of the sleeve is significantly larger than the stress value in the middle of the sleeve. However, with the fracture of the concrete specimen, the force of the specimen on the sleeve becomes uneven, resulting in a decrease in the stress regularity on the sleeve until the end of the sleeve and the specimen no longer interact, and the stress on the sleeve gradually disappears. 

### 4.2. Numerical Simulation Analysis of Concrete Specimen Fracture 

#### 4.2.1. Fracture Characteristics of Uniaxial Impact Compression of Concrete Specimen

**Figure 10:**
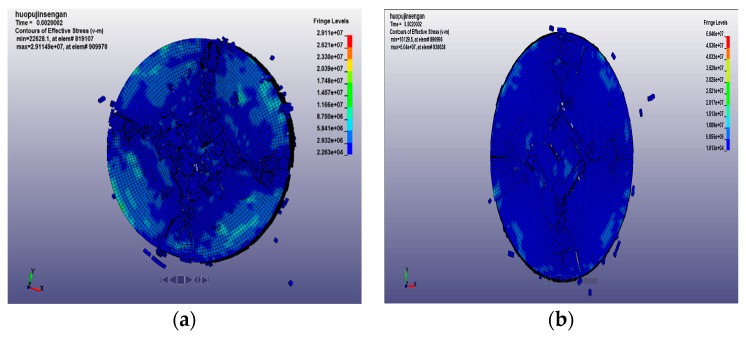
Final fracture morphology of specimen at 20 °C with different loading rates. (**a**) 14.23 m/s; and (**b**) 18.36m/s.

**Figure 11:**
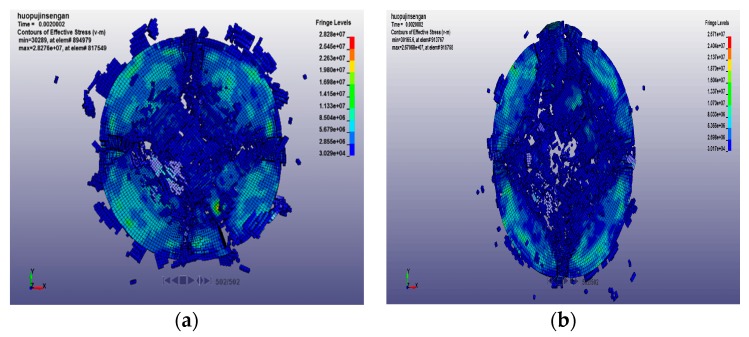
Final fracture morphology of specimen at 400 °C with different loading rates. (**a**) 14.23 m/s; and (**b**) 18.36m/s.

**Figure 12:**
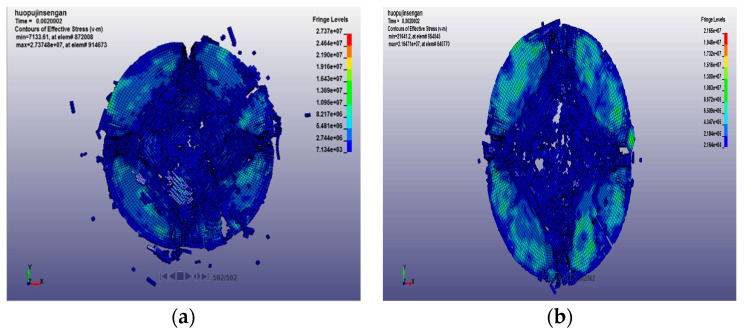
Final fracture morphology of specimen at 600 °C with different loading rates. (**a**) 14.23 m/s; and (**b**) 18.36 m/s.

**Figure 13:**
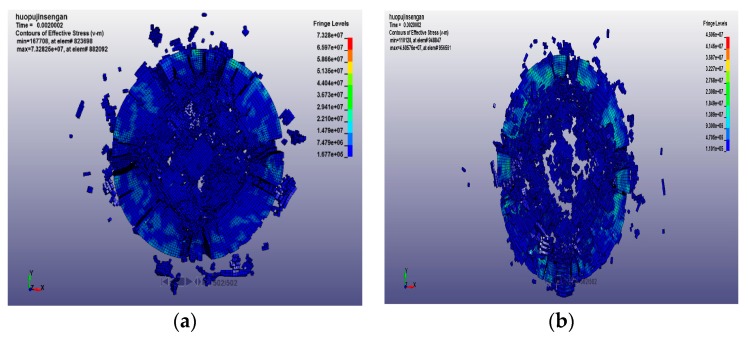
Final fracture morphology of specimen at 800 °C with different loading rates. (**a**) 14.23 m/s; and (**b**) 18.36m/s.

The numerical simulation method can analyze the results that are difficult to obtain in the test, such as the development of cracks, the stress–strain redistribution, and the fracture characteristics of the specimen. The final fracture morphology of the concrete specimens at 20 °C, 400 °C, 600 °C, and 800 °C with loading rates of 14.23 m/s and 18.36 m/s are shown in Figure 10, Figure 11, Figure 12 and Figure 13 respectively. After the specimen is subjected to impact compression, expansion deformation occurs in the radial direction, and the concrete material undergoes radial tensile failure when the ultimate tensile strain value is reached. With the penetration of the split crack, the specimen is divided into several cylinders. The stress and strain on the specimen are redistributed again. The stress gradually decreases to the edge from the center, and the circumferential diffusion characteristics are obvious.

The concrete impact failure process is the propagation process of stress waves from the impact end to another end, and the microcracks expand and merge to form macroscopic cracks, resulting in the final complete destruction of the specimen. It can be seen from the figure that the form of concrete fracture under a low impact rate and a high impact rate is very different. At a low impact rate, the energy generated by the impact is small, the stress level is low, the time for the existing microcracks to expand and grow is relatively long, and the newly generated cracks require more energy, so fewer new cracks appear. Moreover, the mutual influence of microcracks is small, the damage of the specimen is lighter, and the concrete is broken into blocks. However, under the action of a high impact rate, the stress level is high, and many microcracks expand almost at the same time, and the mutual influence between the microcracks is strong. At the same time, a large number of new cracks appeared. Finally, the damage of the specimen is serious, the specimen is broken more thoroughly, and the broken pieces are smaller. At the initial stage of loading, regardless of the impact rate, the fracture of the specimen does not damage at both the front and rear end faces of the specimen, but cracks appear at the side of the specimen, which develop rapidly, eventually causing the sample to break.

At the same loading rate, when the temperature is low, the fracture shape consists of mostly strip-shaped, large, and medium-sized block-shaped fragments. When the temperature is high, it consists of a mostly uniform and fine particle powder. This is because, at low temperatures, the internal crack of the specimen is less, and it spreads directly to the original position under the action of the dynamic load, causing a small degree of fracture and a cleavage-like failure. As the heating temperature increases, the axial and transverse crack distribution increases. Under the action of dynamic load, it loses stability quickly and cuts the specimen into fine particles. The specimen is thus broken and causes the crushing damage.

It can be seen from the above figures and Table 7 that with the increase of the loading rate at the same temperature, the crack initiation time is earlier, and the degree of fragmentation increases at the same loading time. At the same impact rate, the crack initiation time of the concrete specimen increases with the increase of temperature, and the final failure element of the specimen increases.

#### 4.2.2. Fracture Characteristics of Passive Confining Pressure of Concrete Specimens

**Figure 14:**
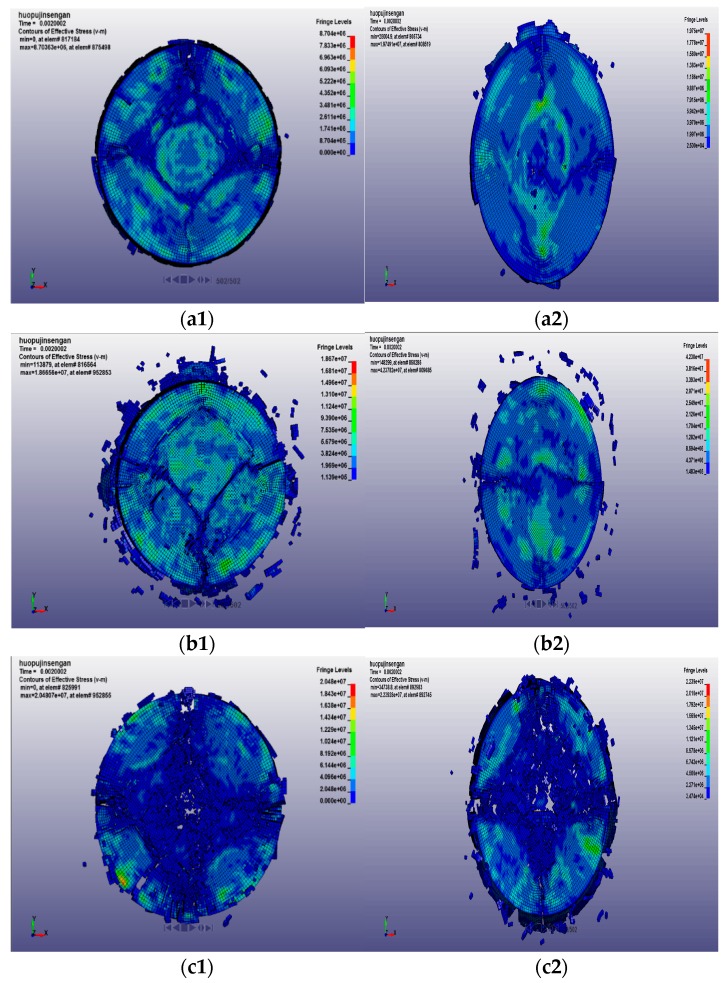

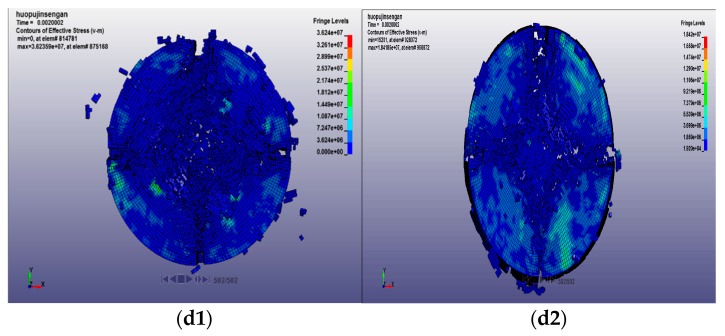
Fracture state of concrete specimen under passive confining pressure at different temperatures. (**a1**) 20 °C, 14.23 m/s; (**a2**) 20 °C, 18.36 m/s; (**b1**) 400 °C, 14.23 m/s; (**b2**) 400 °C, 18.36 m/s; (**c1**) 600 °C, 14.23 m/s; (**c2**) 600 °C, 18.36 m/s; and (**d1**) 800 °C, 14.23 m/s; (**d2**) 800 °C, 18.36 m/s.

Under the condition of passive confining pressure, the crack propagation and cracking state of concrete specimens subjected to impact compression with different loading rates and treatment temperatures are shown in Figure 14.

Under the action of passive confining pressure, it can be seen from the above figure that when the loading rate is low, the range of the confining pressure on the specimen is small, and the degree of fracture is circularly diffused, and the closer to the center of the specimen, the higher the degree of fracture. Since the center of the specimen is far away from the sleeve, it is less affected by the confining pressure constraint, and the edge of the specimen is greatly affected by the confining pressure due to the proximity of the sleeve. Therefore, the confining pressure reduces the degree of fracture of the edge of the specimen and improves the strength of the concrete. When the loading rate is increased, the confining pressure is higher. At this time, the influence range of the confining pressure has spread throughout the specimen. For high temperature-treated concrete specimens, due to excessive temperature damage, the compressive strength is seriously reduced. Therefore, the existence of high confining pressure has a positive impact on the degree of damage of concrete specimens. The concrete specimens are more uniformly destroyed, which enhances the degree of fracture of the specimens, but the degree of fracture still has obvious annular diffusion characteristics. In particular, after a high loading rate impact, the number of the simulated failure units at the center of the circle increased when the specimens were treated at 600 °C and 800 °C, resulting in a blank.

## 5. Conclusions

The effects of loading rate and thermal treatment on concrete strength under uniaxial and passive confining pressures were studied by a combination of tests and numerical simulations. Using numerical simulation to analyze the fracture process and fracture state of the concrete specimen, the conclusions of the research are as follows.

The uniaxial impact compression test shows that the compressive strength of concrete increases with the increase of a certain loading rate at the same temperature. At the same loading rate, the compressive strength of concrete increases slightly with the increase of low heating temperatures. When the temperature exceeds 400 °C, the compressive strength decreases sharply as the temperature increases.

The passive confining pressure test shows that with the increase of the loading rate, the passive confining pressure of the concrete is obviously enhanced, and the increase range is 3.29 MPa to 4.31 MPa. The effect of the thermal treatment temperature on the passive confining pressure is not obvious. The sleeve effect of the passive confining pressure significantly improves the deformability of the concrete and increases the compressive capacity of the concrete by 12% to 41%. However, the increase in the strength of the material with the temperature damage effect is reduced, and is particularly obvious above 600 °C and with a high loading rate.

From numerical simulation, it can be found that the fracture form has a strong rate dependence in the crushing process. When the loading rate is low, the fracture form is that of a cleavage-like failure. As the loading rate increases, the fracture form changes to crush failure. In a uniaxial test, as the impact rate increases, the crack initiation time advances, and the degree of fracture increases at the same loading time. The fracture is from the edge to the center, and the core of the specimen remains intact. In the case with confining pressure, the stress gradually decreases toward the diameter edge at the center, and the damage at the center is obvious.

## Figures and Tables

**Table:** (**a**)

Item	Water	Cement	Coarse Aggregate	Fine Aggregate	Fly Ash	Mineral Powder	Additive
Ingredients (kg)	155	250	1165	670	90	60	10
Proportion	0.62	1.00	4.66	2.68	0.36	0.24	0.04

**Table:** (**b**)

**Sieve Diameter (mm)**	31.5	26.5	19.0	16.0	9.5	4.75	2.36
**Residue on Sieve (%)**	0	3	--	37	--	93	99

**Table:** (**c**)

**Sieve Diameter (mm)**	4.75	2.36	1.25	0.63	0.315	0.16
**Residue on Sieve (%)**	2.8	9.7	21.4	48.1	70.5	99.4

**Table 2:** Confining pressure value under different conditions.

	**Initial Impact Loading Rate of**	
	14.23 m/s	18.36 m/s
**Temperature (°C)**	**Passive Confining Pressure Peak Value (MPa)**	
20	5.01	8.30
400	5.05	8.42
600	4.53	8.78
800	4.55	8.86

**Table 3:** Parameter Part 1.

Temperature(°C)	*ρ* (kg/m3)	*f_c_* (MPa)	*T* (MPa)	*G* (Gpa)
20	2400	34.2	3.6	14.9
400	2380	18.7	2.7	5.3
600	2327	15.5	2.4	3.0
800	2297	7.2	1.7	2.7

**Table 4:** Parameter part 2.

Temperature (°C)	*P_c_* (MPa)	*P_L_* (GPa)	*µ_c_*	*µ_L_*	*K*_1_ (Gpa)	*K*_2_ (Gpa)	*K*_3_ (Gpa)
20	11.4	0.8	0.0010	0.10	85	–171	208
400	6.2	0.8	0.0009	0.12	85	–171	208
600	5.2	0.8	0.0013	0.17	85	–171	208
800	2.4	0.8	0.0007	0.22	85	–171	208

**Table 5:** Parameter part 3.

Temperature(°C)	C	SFMAX	EFMIN	*D*_1_	*D*_2_
20	0.007	7	0.01	0.037	1
400	0.007	7	0.01	0.032	1
600	0.007	7	0.01	0.031	1
800	0.007	7	0.01	0.025	1

**Table 6:** Comparison of numerical simulation and experimental results under different loading rates.

Thermal Treatment Temperature (°C)	Loading Rate (m/s)	Method	Peak Stress (MPa)	Peak Strain	Error of Peak Stress (%)	Error of Peak Strain (%)
20	14.23	Experiment	69.03	0.0092	−1.0	2.0
		Simulation	69.77	0.0090		
	18.36	Experiment	90.88	0.0112	2.0	12.5
		Simulation	89.08	0.0098		
400	14.23	Experiment	74.45	0.0105	1.6	3.8
		Simulation	73.28	0.0101		
	18.36	Experiment	91.92	0.0115	1.1	0
		Simulation	89.04	0.0115		
600	14.23	Experiment	70.67	0.0094	−4.2	−4.3
		Simulation	73.66	0.0098		
	18.36	Experiment	86.70	0.0101	4.6	−1.0
		Simulation	82.74	0.0102		
800	14.23	Experiment	56.17	0.0063	3.2	9.5
		Simulation	54.39	0.0057		
	18.36	Experiment	58.79	0.0075	11.4	−4.0
		Simulation	52.08	0.0078		

**Table 7:** Crack initiation time at different temperatures and loading rates.

Thermal Treatment Temperature (°C)	Loading Rate (m/s)	Crack Initiation Time (μs)
20	14.23	543
	18.36	535
400	14.23	539
	18.36	527
600	14.23	535
	18.36	523
800	14.23	531
	18.36	519
